# Phylogenetic Diversity and Antimicrobial Resistance of *Campylobacter coli* from Humans and Animals in Japan

**DOI:** 10.1264/jsme2.ME18115

**Published:** 2019-03-21

**Authors:** Hiroshi Asakura, Junko Sakata, Hiromi Nakamura, Shiori Yamamoto, Satoshi Murakami

**Affiliations:** 1 Division of Biomedical Food Research, National Institute of Health Sciences Tonomachi 3–25–26, Kawasaki-ku, Kawasaki, Kanagawa 210–9501 Japan; 2 Laboratory of Bacteriology, Department of Microbiology, Osaka Institute of Public Health Nakamichi 1–3–69, Higashinari-ku, Osaka, Osaka 523–0025 Japan; 3 Laboratory of Microbiology, Department of Microbiology, Osaka Institute of Public Health Tojo-cho 8–34, Tennoji-ku, Osaka, Osaka 543–0026 Japan; 4 Department of Animal Hygiene, Tokyo University of Agriculture Funako 1737, Atsugi, Kanagawa 243–0034 Japan

**Keywords:** *Campylobacter coli*, multilocus sequence typing (MLST), antimicrobial resistance (AMR), ST-1562, whole genome sequencing (WGS)

## Abstract

The phylogenetic diversity and antimicrobial resistance (AMR) of *Campylobacter coli* from humans and animals in Japan between 2008 and 2014 were investigated. A total of 338 foodborne campylobacterioses were reported in Osaka, and *C. coli* was isolated from 38 cases (11.2%). In the present study, 119 *C. coli* strains (42 from humans, 25 each from poultry, cattle, and swine, and 2 from wild mallard) were examined by multilocus sequence typing (MLST). MLST assigned 36 sequence types (STs), including 14 novel STs; all human strains and 91% of animal strains (70/77) were assigned to the ST-828 clonal complex. The predominant human ST was ST-860 (18/42, 43%), followed by ST-1068 (8/42, 19%); these STs were also predominant in poultry (ST-860, 9/25, 36%) and cattle (ST-1068, 18/25, 72%). ST-1562 was only predominant in swine (11/25, 44.0%). Swine strains showed the greatest resistance to erythromycin (EM; 92.0%), while EM resistance was only found in 2 out of the 42 human strains examined (4.8%). All EM-resistant swine strains (*n*=15) exhibited a common point mutation in the 23S rRNA sequence (A2085G), and the *tetO* gene was detected in 22 out of the 23 TET-resistant swine strains. A whole genome sequencing analysis of four representative swine ST-1562 strains revealed abundant AMR-associated gene clusters in their genomes, suggesting horizontal gene transfer events during host adaptation. This is the first study to demonstrate the phylogenetic diversity and AMR profiles of *C. coli* in Japan. The present results suggest that poultry and cattle are major reservoirs, improving our knowledge on the epidemiological and ecological traits of this pathogen.

Human campylobacteriosis caused by the two pathogens *Campylobacter jejuni* and *C. coli* is one of the leading causes of bacterial gastroenteritis worldwide (11). Epidemiological surveys indicate that *C. coli* accounts for 9% of human campylobacteriosis in the USA (C.D.C., U.S. 2012. National antimicrobial resistance system, enteric bacteria, human strains final report 2010. www.cdc.gov/narms/pdf/2010-annual-report-narms.pdf) and approximately 7% of that in England and Wales (16). Consequently, the majority of research has focused on the epidemiology of *C. jejuni*, while information on the etiology of human *C. coli* infections remains limited (45). Nevertheless, *C. coli* accounts for 15.3% of human campylobacteriosis in France (43), and human clinical symptoms, primarily diarrhea, abdominal pain, and fever, caused by *C. jejuni* and *C. coli* are generally indistinguishable (44). A link has recently been proposed between *C. coli* enterocolitis and myopericarditis (28). These findings suggest the necessity of acquiring epidemiological information not only for *C. jejuni*, but also *C. coli*.

In wildlife and a wide range of domesticated livestock, such as cattle, sheep, swine, and poultry, carriers of *C. jejuni* and *C. coli* are frequently asymptomatic (19, 37). Swine generally exhibit a higher prevalence of *C. coli* than *C. jejuni* (19), while most other animals carry a higher proportion of *C. jejuni* (31). However, the prevalence rates of *C. jejuni* and *C. coli* in retail swine meats are markedly lower (<0.5%) than those in retail poultry meats (52–90%) (1, 17). This epidemiological background suggests that swine meats are not a major transmission vehicle for human infection. However, insufficient information is currently available to clarify the epidemiology of *C. coli* in swine and humans in Japan.

Since the *Campylobacter* genome is hypervariable due to frequent recombination events (49), a multilocus sequence typing (MLST)-based approach to investigating genetic diversity has advantages for elucidating the etiology of this pathogen. A previous MLST-based study showed that poultry and sheep were the main sources of *C. coli* in Scotland (39). Although we previously reported MLST profiles of *C. jejuni* in Japan (3), no *C. coli* data were available.

When the treatment of human campylobacteriosis is required, quinolones, macrolides, and tetracyclines are the drugs of choice (43). However, recent trends towards increased resistance to these antimicrobials among *Campylobacter* (36, 45, 48, 51) indicate the necessity of monitoring the spatiotemporal and/or host-specific dynamics of the antimicrobial susceptibility of *C. coli* as well as *C. jejuni* to identify targets to prevent antimicrobial resistance (AMR). To date, at least two studies on the prevalence of drug resistance in *C. coli* from swine, cattle, and poultry have been reported in Japan (15, 18); however, limited information is available on human clinical strains.

Given this background, we herein surveyed the occurrence of foodborne campylobacterioses involving *C. jejuni* and *C. coli* in Osaka, Japan between 2008 and 2014. *C. coli* strains from humans and animals were examined to elucidate their phylogenetic diversity and AMR profiles. Using these observations of the phylogenetic differences between swine strains and human clinical strains, together with the acquired drug resistance profiles, we analyzed the genomic traits of representative ST-1562 strains from swine and discussed their associations.

## Materials and Methods

### Isolation procedure of C. coli and culture conditions

Between 2008 and 2014, 42 *C. coli* human clinical strains associated with foodborne infection cases were randomly selected from those occurring in Osaka, Japan (one strain per case was selected from 42 cases in which more than 2 individuals were infected; more detailed information is shown in Table S1). *C. coli* strains from the fecal contents of poultry (*n*=25, from 7 farms A–G), swine (*n*=25, from 6 farms H–M), cattle (*n*=25, from 5 farms N–R), and wild birds (mallard; *Anas platyrhynchos*, *n*=2) were also collected during the same period for use as animal-origin strains at local areas of Japan at which food animals were produced and then sent to areas of consumption, such as Osaka (Table S1 and Fig. S1). Regarding the isolation of *C. coli* from fecal samples of each source, we employed the procedure of ISO 10272-1: 2017 (20) accordingly, except for the use of Skirrow *Campylobacter* selective agars (Oxoid, England, UK) as 2^nd^ selective enrichment media from human clinical specimens. The bacterial strains obtained were routinely grown on Mueller-Hinton (MH) agar or broth (Becton Dickinson, Franklin Lakes, NJ, USA) at 37°C in a humidified CO_2_ AnaeroPack-Microaero gas system (Mitsubishi Gas Chemicals, Tokyo, Japan). The strains obtained were stored in 10% glycerol/tryptic soy broth at −80°C until used.

### MLST analysis

Bacterial genomic DNA was extracted using the DNeasy Genomic Extraction Kit (Qiagen, Hilden, Germany) and stored at −20°C until further use. PCR and cycle sequencing reactions were performed according to the guidelines of the *Campylobacter* MLST database (http://pubmlst.org/campylobacter). We confirmed the absence of non-specific PCR amplicons using a 1% agarose gel. ExoSAP-IT was subsequently used to purify the PCR products (Thermo Fisher Scientific, Carlsbad, CA, USA). In sequencing reactions, we used both DNA strands for each allele with a BigDye Terminator ver. 3.1 Ready Reaction Cycle Sequencing Kit on an ABI3730x DNA Analyzer (Thermo Fisher Scientific). The sequences obtained were assembled using CLC DNA Main Workbench ver. 7.2 equipped with an MLST module (QIAGEN-CLC Bio, Aarhus, Denmark). The consensus sequences for each allele were assigned an allele number, a 7-locus (3,309 bp) sequence type (ST), and a clonal complex (CC) through interrogation of the *Campylobacter* MLST database. Unassigned sequences were deposited into the database to obtain new allele or ST numbers according to the guidelines described on the website. Sequence data for each strain were deposited in the database, and each strain ID is shown in Table S1.

### Phylogenetic analyses

To assess the phylogenetic diversity and evolutionary distance between *C. coli* STs, 7 MLST allelic numbers for 36 STs were subjected to the PHYLOViZ program, as described previously (30). In the evolutionary distance analysis, we set ST-860 as the reference ST because of its high frequency and ancestral position within ST-828CC (41).

### Antibiotic susceptibility tests

Antimicrobial susceptibility tests were conducted using antimicrobial impregnated discs (Oxoid) containing tetracycline (TET; 30 μg), erythromycin (EM; 15 μg), norfloxacin (NFLX; 10 μg), ciprofloxacin (CPFX; 10 μg), ofloxacin (OFLX; 10 μg), nalidixic acid (NA; 30 μg), ampicillin (ABPC; 10 μg), and gentamicin (GEN; 10 μg) according to the standard Kirby-Bauer disc diffusion method (5) and performed according to the recommendations of the Clinical Laboratory Standards Institute (8).

### PCR detection of ermB, tetO, and 23S rRNA mutations

Genomic and plasmid DNAs were extracted from 25 swine-origin strains and TET-resistant strains from humans (*n*=26), poultry (*n*=15), and cattle (*n*=2) with the DNeasy Genomic Extraction Kit and QuickLyse Miniprep Kit (Qiagen), respectively. Purified plasmid DNA was further treated with Plasmid-Safe ATP-Dependent DNase (Lucigen, Middleton, Wisconsin) to remove genomic DNA as described (7). We confirmed the absence of genomic DNA in purified plasmid DNA by PCR using *C. coli aspA* primers (Aspcoli S1: 5′-CAAC TTCAAGATGCAGTACC-3′ and Aspcoli S2: 5′-ATCTGCTAAA GTATGCATTGC-3′) as described on the *Campylobacter* MLST database. The presence of the *ermB* and *tetO* genes, which were responsible for bacterial resistance to EM and TET, respectively, was then examined by PCR, as described previously (47, 51). Point mutations in the 23S rRNA gene were examined by MAMA-PCR (2). The presence of these PCR amplicons was established by loading on agarose gels with ethidium bromide staining. We performed the detection of the *tetO* gene from all selected strains in this section, while the detection of the *ermB* and 23S rRNA mutations was only performed on swine strains.

### Whole genome sequencing (WGS)

Genomic DNA was purified from four representative ST-1562 strains that were positive for *tetO* with the 23S rRNA mutation (A2085G) (CCP005, CCP013, CCP014, and CCP022) by Genomic-tip 100/G in combination with the Genomic DNA buffer set (Qiagen), and was then used to construct libraries with the Ion Xpress Plus Fragment Library kit and Hi-Q View Chef 400 kit using the Ion Chef System (Thermo Fisher Scientific). The libraries on Ion 316 Chip v2 BC were then subjected to Ion semiconductor sequencing with the Ion Hi-Q View Sequencing kit in an Ion PGM instrument (Thermo Fisher Scientific). Sequence data (4,099,697, 2,339,519, 2,783,415, and 2,565,055 reads for CCP005, CCP013, CCP014, and CCP022, respectively) were assembled using CLC Genomic Workbench ver. 9.0 (Qiagen). Totals of 185, 70, 123, and 124 contigs were obtained (>500 bases) for the strains CCP005, CCP013, CCP014, and CCP022 at 516-, 335-, 332-, and 282-fold coverages, respectively. Whole genome shotgun sequence data were deposited at DDBJ/EMBL/GenBank under the accession numbers BHEH01000000 (CCP005), BHEI01000000 (CCP013), BHEJ01000000 (CCP014), and BHEK01000000 (CCP022).

### Data analysis

The detection of open reading frames and the annotation of all draft genomes were automatically performed using the RAST service pipeline (4). IslandViewer 4, which is a computational tool that integrates three different genomic island prediction methods, *i.e*., IslandPick, IslandPath-DIMOB, SIGI-HMM, and Islander (http://www.pathogenomics.sfu.ca/islandviewer/) (6), was used to identify genomic islands. ResFinder (https://cge.cbs.dtu.dk/services/ResFinder) was also used to detect the acquired AMR genes and related chromosomal mutations, as described previously (50). The phylogenetic analysis of the sequenced genomes was performed using the CSI Phylogeny ver. 1.4 program (https://cge.cbs.dtu.dk/services/CSIPhylogeny) (23) with complete *C. coli* genomes deposited in the NCBI database.

## Results

### Occurrence and predictive sources of foodborne *C. coli* infections in Osaka, Japan

According to our epidemiological surveillance in Osaka, Japan between 2008 and 2014, 338 foodborne campylobacterioses were reported (annual case numbers ranged between 14 [2012] and 78 [2014]), among which *C. coli* was detected in 38 cases (11.2%) (Table 1). Although the incidence of foodborne campylobacterioses temporarily declined in 2012 and 2013, it increased again in 2014 (Table 1). Out of 338 cases, causative foods were predictively identified in 298 foodborne cases (88.2%), and 67.1% (200 cases) of these cases were associated with the consumption of poultry meats (Table 1), which accounted for 71.1% (27/38 cases) of *C. coli*-associated cases and 57.7% (173/300 cases) of *C. jejuni*-associated cases (Table 1). Year-by-year comparisons of the causative foods responsible for foodborne campylobacteriosis indicated that poultry meats and cattle meats accounted for 55.3 and 23.6% in 2008–2011, respectively, the means of which were thereafter 83.6 abd 1.6% in 2013–2014, and 82.1 and 5.1% in 2014, respectively (Table 1). Thus, these results indicated that the continuous occurrence of *C. coli* infection was increasingly associated with poultry.

### Summary of MLST profiles of *C. coli* strains from humans and animals

The above-described epidemiological background prompted us to characterize *C. coli* as well as *C. jejuni* in Japan by MLST because this approach has the potential to clarify phylogenetic diversity (3, 39). A total of 119 *C. coli* strains from humans (*n*=42, each of which originated from an independent foodborne infection case) and animals (*n*=25 each for cattle, poultry, and swine, plus two from the mallard, *Anas platyrhynchos*, obtained from 10 prefectures; Fig. S1, Table S1), collected between 2008 and 2014, were subjected to the MLST analysis targeting seven genes (*aspA*, *glnA*, *gltA*, *glyA*, *pgm*, *tkt*, and *uncA*). In total, 104 out of the 119 strains (87.4%) were classified into 22 STs, among which ST-860, ST-1068, and ST-1562 accounted for 22.7% (*n*=27), 21.9% (*n*=26), and 9.2% (*n*=11) of the identified strains, respectively. All but one (ST-1243, *n*=1) of the STs in the identified strains belonged to ST-828CC. The remaining 15 strains were assigned to 14 novel STs (ST-8795 to 8809), eight of which (ST-8795 to 8798, 8803, 8804, 8806, and 8807) were assigned to ST-828CC (Tables 2 and S1).

### AMR of *C. coli* strains

A previous study reported that *C. coli* from humans showed greater resistance to multiple antimicrobials than *C. jejuni* (34); therefore, we examined the AMR profiles of these Japanese strains. Human clinical strains showed relatively high resistance to fluoroquinolones (FQs), such as NFLX (64.3% [27/42]), OFLX (57.1% [24/42]), and CPFX (59.5% [25/42]), along with NA (69.0% [29/42]), TET (61.9% [26/42]), ABPC (23.8% [10/42]), and EM (4.8% [2/42]), respectively (Tables 2 and S1). Among the *C. coli* strains from animal sources, poultry strains exhibited greater resistance to FQs (72.0%, 18/25) than human strains (Tables 2 and S1). Swine strains exhibited greater resistance to EM (60.0%, 15/25), TET (92.0%, 23/25), and ABPC (92.0% [23/25]) than strains derived from other sources, while FQ resistance was only found in 3 strains (12.0%) (Tables 2 and S1). Strains of cattle and wild bird origins showed relatively high susceptibilities to the antimicrobials tested (Tables 2 and S1). Thus, these results indicated source-specific variations in AMR profiles in *C. coli*.

### Host-specific phylogenetic diversity and AMR profiles of *C. coli*

A series of host-associated lineages have been reported in *C. jejuni* (3, 38, 39). Therefore, the *C. coli* MLST profiles of each host were comparatively examined using the PHYLOViZ program (30) to indicate population structures and possible evolutionary relationships between the *C. coli* strains from each source. The AMR profiles in each host were also comparatively studied.

#### (i) Human clinical strains

**Fig. 1:**
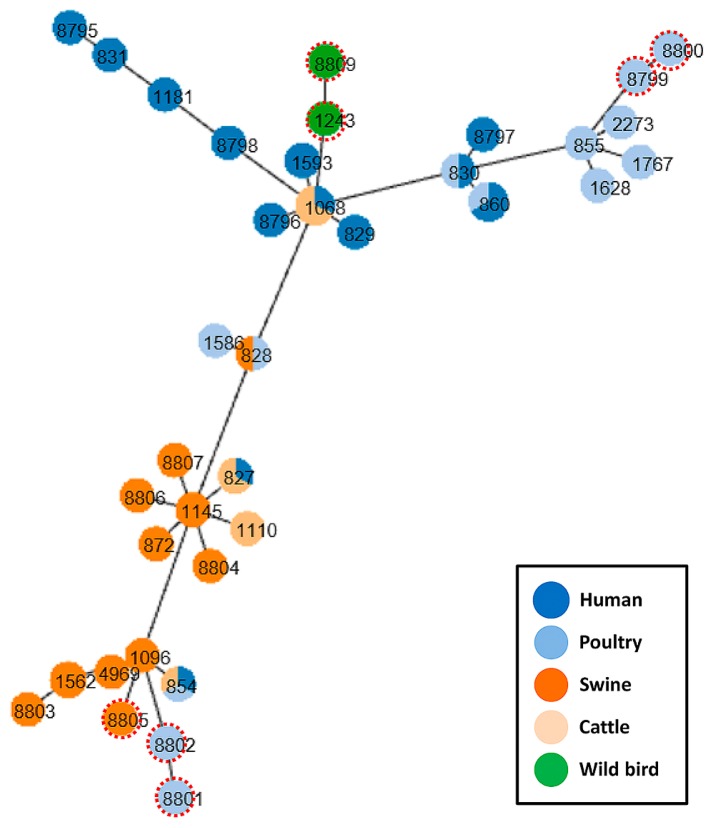
Phylogenetic tree of *C. coli* strains from humans and animals used in the present study. Numbers represent sequence types (STs). Sources of different strains are shown in different colors (humans, dark blue; poultry, light blue; swine, dark orange; cattle, light orange; wild birds, green). STs that were not assigned to ST-828CC are shown with red dotted enclosures.

All human clinical strains (*n*=42) were classified into 13 STs within ST-828CC, of which ST-827, ST-854, ST-860, and ST-1068 demonstrated multiple host associations (Fig. 1). Human strains showed mean distances from ST-860 ranging between 0.00 (ST-860) and 0.71 (ST-1181, ST-831, and ST-8795) (Table 2). ST-860 accounted for 42.9% (18/42) of human strains, followed by ST-1068 (8/42, 19.0%) and ST-1593 (4/42, 9.5%) (Table 2). The resistances of the three dominant STs to NFLX, TET, and ABPC were 78% (*n*=14), 75% (*n*=6), and 100% (*n*=4) (for NFLX); 72% (*n*=13), 88% (*n*=7), and 50% (*n*=2) (for TET); and 33% (*n*=6), 25% (*n*=2), and 25% (*n*=1) (for ABPC) (Table 2).

#### (ii) Poultry strains

The 25 poultry strains were genotyped into 14 STs, of which 21 strains in 10 STs were classified into ST-828CC (Table 2). Similar to the human strains, within ST-828CC, ST-860 was predominant (9/25, 36.0%), followed by ST-1767 (4/25, 16.0%) (Table 2). Among the poultry-origin STs, four STs (ST-828, ST-830, ST-854, and ST-860) were also found in other hosts (human or cattle) (Table 2, Fig. 1). Four STs that were not assigned to any CC (STs-8799, 8800, 8801, and 8802) were similarly distant (mean distances ranging between 0.57 and 0.86) from the ancestral ST-860, in contrast to the three predominant STs (ST-860, ST-1767, and ST-1628; mean distances of 0.00 to 0.29) (Table 2, Fig. 1). Among the three predominant STs, ST-860 showed 100% NFLX and TET resistance (9/9), while all ST-1767 strains (4/4) were sensitive to NFLX (Tables 2 and S1).

#### (iii) Swine strains

Swine strains (*n*=25) were genotyped into 11 STs, and one ST (ST-8805) was not assigned to ST-828CC (mean distance from ST-860, 0.71; Table 2). ST-1562 and ST-1145 accounted for 44.0% (*n*=11, mean distance 0.57) and 12.0% (*n*=3, mean distance 0.43), respectively (Table 2, Fig. 1). ST-828 was also found in poultry, but no other STs from swine were detected from other sources (Table 2, Fig. 1). The ST-1562 strains exhibited resistance to EM (72.7%; 8/11), TET (90.9%, 10/11), and ABPC (90.9%, 10/11) (Table 2). Strains with the second most common sequence type, ST-1145, showed 100% resistance to TET and ABPC (3/3), but were susceptible to EM (Table 2).

#### (iv) Cattle strains

The 25 cattle strains were genotyped into only four STs (ST-1068, ST-827, ST-1110, and ST-854), and all belonged to ST-828CC even though they originated from cattle farmed at five different locations (Table S1, Fig. S1). ST-1068 and ST-827 accounted for 72.0% (*n*=18) and 16.0% (*n*=4) (both mean distances from ST-860 at 0.43) of the cattle strains, and were also detected in human strains at 19.0% (8/42) and 4.8% (2/42), respectively (Table 2, Fig. 1). Among these, only ST-1068 exhibited resistance to NFLX (16.7%, 3/18) and TET (11.1%, 2/18), while the remaining cattle strains, classified as ST-827, ST-1110, or ST-854, were sensitive to all antimicrobials tested (Tables 2 and S1).

#### (v) Wild bird strains

The two wild mallard strains were classified as ST-1243 and ST-8809, with no assignment to any CC (Tables 2 and S1). Their phylogenetic distances from ST-860 were 0.86 and 1.00 because they were the most distant lineages among the strains tested (Table 2, Fig. 1). These two strains were sensitive to all antimicrobials, except ABPC in the ST-1243 strain (Tables 2 and S1).

Collectively, these results showed that certain poultry and cattle strains (*i.e*., ST-860 and ST-1068) phylogenetically and phenotypically overlapped with human strains, whereas swine strains were evolutionarily distant from human clinical strains.

### Genetic and genomic features of AMR profiles in TET- and/or EM-resistant strains

Since swine strains exhibited increased resistance to EM and TET (Table 3) with a distant phylogeny from human strains (Fig. 1), we examined the presence of *tetO* (for TET resistance) in the swine strains (*n*=25) together with the TET-resistant strains from humans (*n*=26), poultry (*n*=15), and cattle (*n*=2) by a PCR detection assay. The results obtained revealed that all 15 EM-resistant strains showed a common mutation in domain V of 23S rRNA (A2085G); the *tetO* gene was also detected in the chromosomal DNA of 22 TET-resistant strains, and one strain was *tetO*-positive from plasmid DNA (CCP012) (Table 3). Comparatively, most of the *tetO* genes detected in TET-resistant strains from humans, poultry, and cattle strains were located on plasmids, except for one poultry strain (Table 3). Consistently, most of the TET-resistant swine strains (21/25, except for four strains CCP010, CCP012, CCP017, and CCP021, which possessed plasmids ranging between 0.9 and 32.0 kb) possessed no plasmids, whereas all of the TET-resistant strains from humans or poultry harbored plasmids (Tables 3 and S1). We simultaneously examined the prevalence of *ermB*, the 23S rRNA mutation (for EM resistance) in swine strains, by the PCR detection assay and the results obtained showed that no *ermB* genes were present in any of these strains (Table 3).

In order to examine AMR-associated genomic features in swine strains that showed phylogenetical and phenotypical dissimilarities to *C. coli* strains from humans, poultry, and cattle, we selected four representative ST-1562 strains (CCP005, CCP013, CCP014, and CCP022), and subjected them to a WGS analysis because this genotype was predominant among swine strains and exhibited resistance to EM, TET, and ABPC (Tables 2 and 3). The results obtained revealed the common distribution of a set of AMR-associated genes, such as *tetO*, *bla*_OXA-61_ (the β-lactamase class D gene, for ABPC resistance), and *aph*(3′)-III (encoding aminoglycoside 3′-phosphotransferase, for kanamycin (KM) resistance), in their genomes (Table S2). The IslandViewer 4 program further revealed that a genomic island cluster harboring *tetO* (for CP0013)-*hpt* (hygromycin B-phosphotransferase)-*ant* (6)-I (aminoglycoside 6-nucleotidyltransferase for resistance to aminoglycosides, such as KM)-*sat4* (streptothricin acetyltransferase)-*aph*(3′)-III (aminoglycoside 3′-phosphotransferase)-*tetO* (for CP0005) genes conferred multidrug resistance in strains CCP005 and CCP013 (Table S2). Likewise, the CCP014 and CCP022 strains both harbored multidrug resistance gene clusters, consisting of *tetO*-*hpt*-*cat* (chloramphenicol O-acetyltransferase)-*aph*(3′)-III and *cat*-*aph*(3′)-III, respectively (Table S2). However, their actual AMR phenotypes were restricted to resistance to TET, ABPC, and EM (Table S2).

**Fig. 2:**
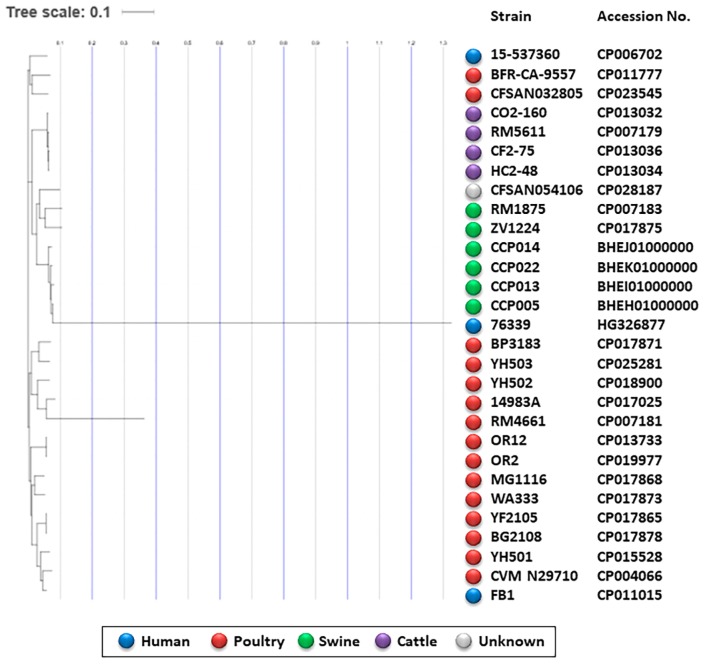
Phylogenetic tree of *C. coli* strains based on a whole genome single nucleotide polymorphism analysis using the CSI Phylogeny 1.4 program. Four representative *C. coli* ST-1562 strains from swine are comparatively analyzed with 24 *C. coli* complete genomes deposited on the NCBI database (accession numbers are shown). Sources of different strains are shown in different colors (humans, blue; poultry, red; swine, green; cattle, purple; unknown, grey).

A single nucleotide polymorphisms-based phylogenomic tree analysis showed that these ST-1562 strains were grouped into one cluster together with two swine strains, RM1875 and ZV1224, and one human strain, 76339 (Fig. 2). They were distant from one cluster, which contained strains of poultry- or human-origin (*i.e*. BP3183) (Fig. 2), indicating that swine-associated *C. coli* has an altered genomic backbone. Collectively, these results demonstrated that the ST-1562 strains dominant in the swine population frequently gained resistance to TET/EM/ABPC through the acquisition of multiple antibiotic resistance genes on their genomes and had a genomic background dissimilar to that of most human clinical strains.

## Discussion

We herein investigated the phylogenetic diversity and AMR profiles of 119 *C. coli* strains from humans and animals collected between 2008 and 2014 in Osaka, Japan. Epidemiological surveillance has indicated that poultry and cattle meats are one of the major sources of human infection. In the present study, phylogenetic and AMR data supported this finding and revealed that swine may exhibit a weaker association with human infection as well as harboring multidrug resistance gene clusters. Furthermore, representative multidrug resistant strains of ST-1562, a predominant genotype in swine, phenotypically and phylogenetically dissimilar to human strains were selected as targets for a WGS analysis to investigate the genomic features associated with multidrug resistance phenotypes.

*Campylobacter* is a rapidly evolving bacterial genus with a massive recombination potential that may generate niche-specific genotypes. Thus, these pathogens may exhibit increased genome sequence diversity among strains, thereby affecting host adaptation (41), cell morphology (12), and biofilm formation (35). The MLST approach has helped attribute the sources of infection by exploring differences in the frequencies of *Campylobacter* STs that inhabit different animal and environmental reservoirs (26). Among the many MLST profiles from Western countries, ST-828CC has been recognized as one of the major CCs representing human *C. coli* infection (21, 40). Therefore, the present results provide evidence of a similar phylogenetic distribution of this pathogen in Japan, despite geographical differences.

A host-by-host phylogenetic comparison revealed the overlapping distributions of the ST-860 and ST-1068 strains between poultry and humans and between cattle and humans, respectively, with multiple numbers of isolates. According to the *Campylobacter* MLST database, a total of 118 ST-860 strains (including 27 strains in the present study) have been deposited, and all of them originated either from humans or poultry (as of 29 July 2018), suggesting that ST-860 exhibits greater fitness in poultry hosts than in cattle and swine, thereby linking these strains to human infection. The frequent detection of ST-860 from humans was reported in Luxemburg (29). These findings demonstrate the necessity for continuous epidemiological surveillance, particularly in humans and poultry.

The other human-associated strain, ST-1068, was identified as a cattle-adapted genotype in the USA (27). This genotype was detected from humans between 2008 and 2011; however, it was not detected in 2012 or after (Table S1). One explanation for this spatiotemporal shift may be the Japanese government adopting two hygienic control measures focusing on the consumption of raw cattle meat and liver in 2011 and 2012, respectively, to prevent life-threatening infections with enterohemorrhagic *Escherichia coli* (EHEC) through the raw consumption of these meats (Food safety commission of Japan. Risk assessment reports of EHEC and *Salmonella* species in cattle meats for raw consumption (www.fsc.go.jp/sonota/emerg/namaniku_hyoka_gaiyo_english.pdf) and of EHEC in cattle liver for raw consumption (www.fsc.go.jp/fsciis/evaluationDocument/show/kya20120409458)). It is important to note that these hygienic control measures may influence the incidence of foodborne campylobacteriosis by cattle meat and liver because cattle-associated campylobacteriosis decreased its yields after 2011 (Table 1), which, in turn, proposes the necessity of regulating the contamination risks of *C. jejuni* and *C. coli* in poultry meats throughout the food chain.

As other STs showing overlapping distributions between multiple hosts, ST-827 and ST-854 were detected herein. However, based on the MLST database, it is likely that these STs were distributed in multiple host species based on the MLST database, suggesting their ecological features as generalists.

Among swine strains, ST-1562 was the predominant ST, with the highest frequency of multidrug resistance (11/25); however, this ST has not been detected from humans, even in the MLST database (as of July 31, 2018). Genomic and genetic approaches provided one explanation for the predominance and acquired AMR of this genotype from swine because these strains harbored a point mutation in 23S rRNA (A2075G) in combination with the presence of AMR-associated genes (*i.e*., *tetO*). Previous studies demonstrated that poultry-associated *C. jejuni* frequently harbored the *tetO* gene on their plasmids (3, 9), and these *tetO*-mediated plasmids effectively increase genomic plasticity in *Campylobacter* (14). In support of this, the present results demonstrated that most of the TET-resistant human, poultry, and cattle strains harbored the *tetO* gene on their plasmids, while swine strains harbored it on chromosomes. Further studies are required to elucidate these differential distribution patterns of the *tetO* gene in *C. coli* by source animals. A more detailed sequence feature study of swine-originating AMR *C. coli* on both chromosomes and plasmids may clarify the transfer efficiency of AMR-related genes on plasmids or possible integration into chromosomes.

In Japan, annual sales volumes of TET and related antimicrobials in 2014 for swine, cattle, and poultry were 239,076, 18,323, and 23,124 kg, respectively (Ministry of Agriculture, Forestry and Fisheries (MAFF), Japan; http://www.maff.go.jp/nval/iyakutou/hanbaidaka/), indicating its greatest use for swine. The use of large amounts of TET for swine may be associated with the chromosomal acquisition of the *tetO* gene in *C. coli* with altered interactions with intestinal microbiota in the swine host.

Swine strains also exhibited greater EM resistance profiles than *C. coli* from other sources. Annual sales volumes of EM in 2014 for swine, cattle, and poultry were 12.8, 1.4, and 0 kg, respectively, based on the above-described data by MAFF, Japan, indicating that swine was the main target for its use, similar to TET. In addition, tylosin (a macrolide) has been used as a growth promoter in swine, but not in poultry, which may explain the selection of EM resistance in swine strains in Japan. Although it currently remains unclear whether the use of tylosin affects EM resistance in *Campylobacter* spp., a previous study demonstrated that *Enterococcus* spp. isolated from swine fed in farms with the use of tylosin exhibited higher rates of EM resistance than those from swine fed without its use in the USA (22). Since the European Union banned the use of tylosin as a growth promoter in animal feeds from January 1999, the establishment of control measures in swine may restrict EM resistance in *C. coli* from swine.

A WGS analysis revealed that a series of multidrug resistance gene clusters, including the *tetO* gene, were distributed in swine ST-1562 strains, and these clusters showed partial sequence similarities (*i.e*., *ant*[6]-I=>*sat4*=>*aph*[3′]-III) to the plasmid pRE25 of *E. faecalis* (accession No. X92945), which was initially detected in raw-fermented swine sausage (46). *E. faecalis* of swine origin frequently harbors mosaic plasmids containing the *Tn1546* backbone (13). Thus, these plasmids in the swine intestinal microbiota may mediate horizontal gene transfer (including that of drug resistance genes) to *C. coli*. Meanwhile, the *ant*-like gene appears to be widespread in hypervariable regions of the *C. coli* genome regardless of the host and environment (33). Several AMR genes (*i.e*., ant [6]-I in the CCP005 strain) were cryptic without expressing AMR phenotypes (Table S2). Further studies focusing on the genomic plasticity of swine-adapted *C. coli* may contribute to elucidating the role of the chromosomal *ant*-like gene in the process of host adaptation in this pathogen.

In wild birds, *C. jejuni* showed the highest prevalence, while limited numbers of *C. coli* and *C. lari* were present (24, 25, 32, 42), and 20 *C. coli* isolates from wild birds in South Korea were susceptible to all antimicrobials tested (25). Additionally, Okamura and co-workers demonstrated a distant phylogenetic relationship of wild crow *C. jejuni* with those from poultry in Japan (32). Further studies are required to confirm that *C. coli* strains of wild bird origin are more weakly associated with human infection than those from poultry and ruminants. In conclusion, the present study is the first to demonstrate that almost all foodborne *C. coli* infections in Osaka, Japan, were caused by ST-828CC and that poultry and cattle hosts exhibit increased associations with human infection in Osaka, Japan. The common *C. coli* strains that infect humans may be adapted to a generalist lifestyle, permitting rapid transmission between different hosts (10). Continuous studies using WGS technology will improve our understanding of the sources of infection and corroborate the occurrence and transmission of AMR and bacterial host adaptation mechanisms.

## Supplementary Information







## Figures and Tables

**Table 1:** Incidence of foodborne campylobacteriosis reported in Osaka, Japan between 2008 and 2014.

Causative pathogen/food*1	2008	2009	2010	2011	2012	2013	2014	2008–2014
*C. jejuni*	53	32	39	55	12	44	65	300
Poultry meat	26	19	17	36	7	39	55	173
Cattle meat	15	6	16	8	1	0	2	33
Others*2	3	3	2	2	0	0	2	9
Unknown	9	4	4	9	4	5	6	32
*C. coli*	0	3	1	2	1	1	5	13
Poultry meat	0	1	1	2	1	1	3	9
Cattle meat	0	0	0	0	0	0	1	1
Others	0	0	0	0	0	0	0	0
Unknown	0	2	0	0	0	0	1	3
*C. jejuni* and C. coli*3	4	4	2	4	1	2	8	25
Poultry meat	1	2	2	3	1	2	6	18
Cattle meat	1	1	0	0	0	0	1	3
Others	0	0	0	0	0	0	0	0
Unknown	2	1	0	1	0	0	1	5

Total	57	39	42	61	14	47	78	338

*1 Food categories shown represent predicted causative foods from which *C. jejuni*, *C. coli* or both bacteria are detected.

*2 Others include swine meat, mallard meat, and salad with smoked poultry meat.

*3 Foodborne cases in which *C. jejuni* and *C. coli* were both simultaneously detected.

**Table 2:** Summary of MLST and AMR profiles of *C. coli* strains from humans and animals in Japan.

Source	No. isolate	Clonal Complex*1 (No. isolate)	ST*2	No. isolate	Antimicrobial resistance to*3								Evolutional distance*4	
	
					TET	EM	NFLX	OFLX	CPFX	NA	ABPC	GM	Node	Distance
Human	42	ST-828CC (42)	860	18	13 (72%)	0 (0%)	14 (78%)	12 (67%)	12 (67%)	16 (89%)	6 (33%)	0 (0%)	0	0.00
			1068	8	7 (88%)	0 (0%)	6 (75%)	6 (75%)	6 (75%)	6 (75%)	2 (25%)	0 (0%)	3	0.43
			1593	4	2 (50%)	0 (0%)	4 (100%)	3 (75%)	4 (100%)	4 (100%)	1 (25%)	1 (25%)	4	0.57
			827	2	1 (50%)	0 (0%)	0 (0%)	0 (0%)	0 (0%)	0 (0%)	1 (50%)	0 (0%)	3	0.43
			829	2	0 (0%)	0 (0%)	2 (100%)	2 (100%)	2 (100%)	2 (100%)	0 (0%)	0 (0%)	2	0.29
			830	1	1 (100%)	0 (0%)	1 (100%)	1 (100%)	1 (100%)	1 (100%)	0 (0%)	0 (0%)	1	0.14
			1181	1	0 (0%)	0 (0%)	0 (0%)	0 (0%)	0 (0%)	0 (0%)	0 (0%)	0 (0%)	5	0.71
			831	1	0 (0%)	0 (0%)	0 (0%)	0 (0%)	0 (0%)	0 (0%)	0 (0%)	0 (0%)	5	0.71
			854	1	0 (0%)	0 (0%)	0 (0%)	0 (0%)	0 (0%)	0 (0%)	0 (0%)	0 (0%)	4	0.57
			**8795**	1	1 (100%)	1 (100%)	0 (0%)	0 (0%)	0 (0%)	0 (0%)	0 (0%)	0 (0%)	5	0.71
			**8796**	1	1 (100%)	1 (100%)	0 (0%)	0 (0%)	0 (0%)	0 (0%)	0 (0%)	0 (0%)	3	0.43
			**8797**	1	0 (0%)	0 (0%)	0 (0%)	0 (0%)	0 (0%)	0 (0%)	0 (0%)	0 (0%)	3	0.43
			**8798**	1	0 (0%)	0 (0%)	0 (0%)	0 (0%)	0 (0%)	0 (0%)	1 (100%)	0 (0%)	4	0.57

Poultry	25	ST-828CC (21)	860	9	9 (100%)	0 (0%)	9 (100%)	9 (100%)	9 (100%)	9 (100%)	0 (0%)	0 (0%)	0	0.00
			1767	4	0 (0%)	0 (0%)	0 (0%)	0 (0%)	0 (0%)	0 (0%)	0 (0%)	0 (0%)	1	0.14
			1628	2	1 (50%)	0 (0%)	1 (50%)	1 (50%)	1 (50%)	1 (50%)	0 (0%)	0 (0%)	2	0.29
			828	1	0 (0%)	0 (0%)	1 (100%)	1 (100%)	1 (100%)	1 (100%)	0 (0%)	0 (0%)	3	0.43
			830	1	0 (0%)	0 (0%)	1 (100%)	1 (100%)	1 (100%)	1 (100%)	0 (0%)	0 (0%)	1	0.14
			854	1	0 (0%)	0 (0%)	1 (100%)	1 (100%)	1 (100%)	1 (100%)	0 (0%)	0 (0%)	4	0.57
			855	1	1 (100%)	0 (0%)	1 (100%)	1 (100%)	1 (100%)	1 (100%)	0 (0%)	0 (0%)	2	0.29
			1586	1	1 (100%)	0 (0%)	1 (100%)	1 (100%)	1 (100%)	1 (100%)	0 (0%)	0 (0%)	3	0.43
			2273	1	1 (100%)	0 (0%)	1 (100%)	1 (100%)	1 (100%)	1 (100%)	0 (0%)	0 (0%)	3	0.43

		UA (4)	**8799**	1	0 (0%)	0 (0%)	0 (0%)	0 (0%)	0 (0%)	0 (0%)	0 (0%)	0 (0%)	4	0.57
			**8800**	1	0 (0%)	0 (0%)	0 (0%)	0 (0%)	0 (0%)	0 (0%)	0 (0%)	0 (0%)	5	0.71
			**8801**	1	1 (100%)	0 (0%)	1 (100%)	1 (100%)	1 (100%)	1 (100%)	0 (0%)	0 (0%)	6	0.86
			**8802**	1	1 (100%)	0 (0%)	1 (100%)	1 (100%)	1 (100%)	1 (100%)	0 (0%)	0 (0%)	4	0.57

Swine	25	ST-828CC (24)	1562	11	10 (91%)	8 (73%)	0 (0%)	0 (0%)	0 (0%)	0 (0%)	10 (91%)	0 (0%)	4	0.57
			1145	3	3 (100%)	0 (0%)	0 (0%)	0 (0%)	0 (0%)	0 (0%)	3 (100%)	0 (0%)	3	0.43
			872	2	2 (100%)	2 (100%)	0 (0%)	0 (0%)	0 (0%)	0 (0%)	2 (100%)	0 (0%)	2	0.29
			**8807**	2	2 (100%)	2 (100%)	0 (0%)	0 (0%)	0 (0%)	0 (0%)	2 (100%)	0 (0%)	3	0.43
			1096	1	1 (100%)	0 (0%)	1 (100%)	1 (100%)	1 (100%)	1 (100%)	1 (100%)	0 (0%)	4	0.57
			828	1	1 (100%)	0 (0%)	0 (0%)	0 (0%)	0 (0%)	0 (0%)	0 (0%)	0 (0%)	3	0.43
			4969	1	0 (0%)	0 (0%)	0 (0%)	0 (0%)	0 (0%)	0 (0%)	1 (100%)	0 (0%)	4	0.57
			**8803**	1	1 (100%)	0 (0%)	0 (0%)	0 (0%)	0 (0%)	0 (0%)	1 (100%)	0 (0%)	4	0.57
			**8804**	1	1 (100%)	1 (100%)	1 (100%)	1 (100%)	1 (100%)	0 (0%)	1 (100%)	0 (0%)	5	0.71
			**8806**	1	1 (100%)	1 (100%)	0 (0%)	0 (0%)	0 (0%)	0 (0%)	1 (100%)	0 (0%)	3	0.43

		UA (1)	**8805**	1	1 (100%)	1 (100%)	1 (100%)	1 (100%)	1 (100%)	0 (0%)	1 (100%)	0 (0%)	5	0.71

Cattle	25	ST-828CC (25)	1068	18	2 (11%)	0 (0%)	3 (17%)	3 (17%)	3 (17%)	0 (0%)	0 (0%)	0 (0%)	3	0.43
			827	4	0 (0%)	0 (0%)	0 (0%)	0 (0%)	0 (0%)	0 (0%)	0 (0%)	0 (0%)	3	0.43
			1110	2	0 (0%)	0 (0%)	0 (0%)	0 (0%)	0 (0%)	0 (0%)	0 (0%)	0 (0%)	3	0.43
			854	1	0 (0%)	0 (0%)	0 (0%)	0 (0%)	0 (0%)	0 (0%)	0 (0%)	0 (0%)	4	0.57

Wild bird	2	UA (2)	1243	1	0 (0%)	0 (0%)	0 (0%)	0 (0%)	0 (0%)	0 (0%)	1 (100%)	0 (0%)	6	0.86
			**8809**	1	0 (0%)	0 (0%)	0 (0%)	0 (0%)	0 (0%)	0 (0%)	0 (0%)	0 (0%)	7	1.00

*1 UA, Unassigned to any CCs.

*2 Novel STs are shown in bold.0

*3 No. (percentage) of isolates resistant to the three representative antimicrobials are shown.

Intermediate (I) data are omitted from this table. More detailed information is available in Table S1.

*4 Evolutional distance is calculated by the PHYLOViZ program based on MLST data with ST-860 as a reference (distance mean=0.00).

**Table 3:** PCR detection of *ermB*, 23S rRNA mutation, and *tetO* gene in *C. coli* from swine and of *tetO* gene in TET-resistant *C. coli* from other sources.

Host	AMR profile (EM/TET)*1	No. strain	ST (No. strain)	No. plasmid-positive strain	No. positive for			

					*ermB*	23S rRNA (A2075G)	*tetO* (on chromosome)	*tetO* (on plasmid)
Swine	EM^R^, TET^R^	15	1562 (8)	2	0	7	6	1
			872 (2)	0	0	2	2	0
			8804 (1)	0	0	1	1	0
			8805 (1)	0	0	1	1	0
			8806 (1)	0	0	1	1	0
			8807 (2)	1	0	2	2	0

	EM^S^, TET^R^	8	1562 (2)	0	0	0	3	0
			1145 (3)	0	0	0	3	0
			8803 (1)	0	0	0	1	0
			828 (1)	1	0	0	1	0
			1096 (1)	0	0	0	1	0

	EM^S^, TET^S^	2	1562 (1)	0	0	0	0	0
			4969 (1)	0	0	0	0	0

Human	TET^R^	26	860 (13)	12	NT*2	NT	1	12
			1068 (7)	7	NT	NT	0	7
			1593 (2)	1	NT	NT	0	1
			827 (1)	1	NT	NT	0	1
			830 (1)	1	NT	NT	0	1
			8795 (1)	1	NT	NT	0	1
			8796 (1)	1	NT	NT	0	1
	
Poultry		15	860 (9)	8	NT	NT	1	8
			855 (1)	1	NT	NT	0	1
			1586 (1)	1	NT	NT	0	1
			1628 (1)	1	NT	NT	0	1
			2273 (1)	1	NT	NT	0	1
			8801 (1)	1	NT	NT	0	1
			8802 (1)	1	NT	NT	0	1
	
Cattle		2	1068 (2)	2	NT	NT	0	2

*1 EM, erythromycin; TET, tetracycline; R, resistant; S, sensitive.

*2 NT, not tested.
