# Supplementary material for: Phylogenetic Diversity and Antimicrobial Resistance of Campylobacter coli from Humans and Animals in Japan
Source: Microbes Environ. 2019 Mar 21;34(2):146–54. doi: 10.1264/jsme2.ME18115 (PMC6594732; doi:10.1264/jsme2.ME18115)
Supplement: Supplementary file 1 [file 34_146_s1.pdf]

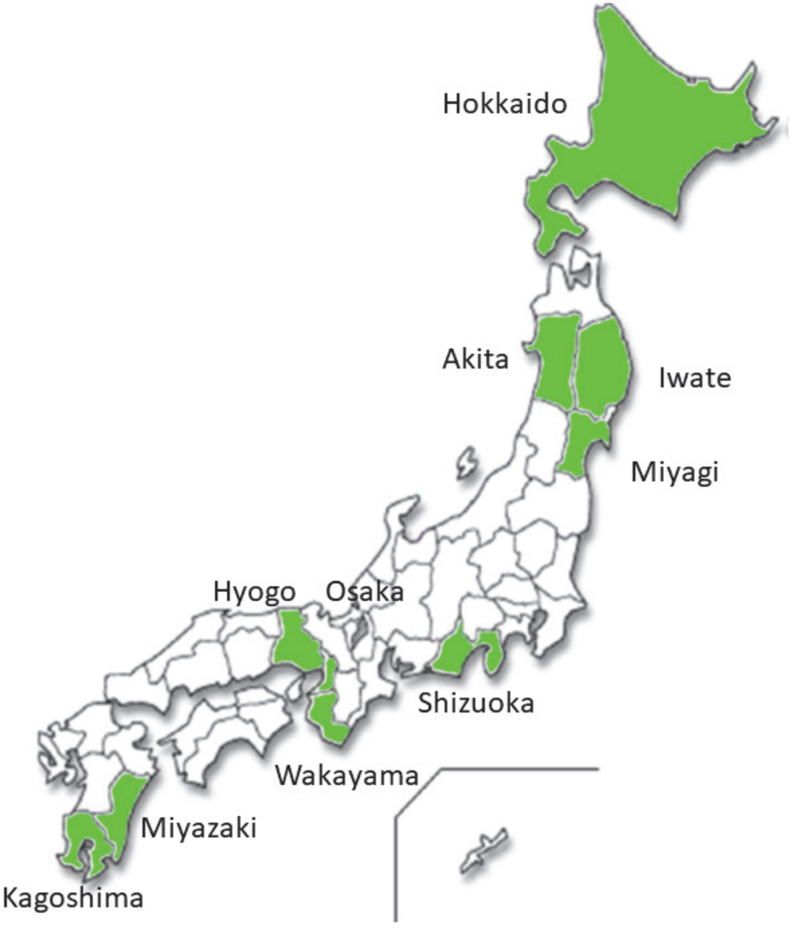

Fig. S1. Geographical map of Japan indicating the locations where *C. coli* was collected (green coloured). More detailed information for each strain is shown in Table S1.
